# Skin Diseases and Syphilis

**Published:** 1895-01

**Authors:** 


### *SELECTIONS AND ABSTRACTS.*

### SKIN DISEASES AND SYPHILIS.

EDITED BY DR. M. B. HUTCHINS.

ACCARAIN (*Jour. des Mal. Cut. et de Syph.*, 1893, p. 673) reports a case of syphilis communicated by means of a razor.—*M. für Prak. Derm.*, XIX., No. 5.

[Various contagious diseases may be acquired in the barber-shop, as the cleansing to which the utensils are subjected certainly does not sterilize them.—ED.]

### A NEW METHOD OF TREATING LATE SYPHILITIC LESIONS.

Peroni says many cases which are not influenced by iodine and mercury can be cured by the local application of ichthyol. In other cases calomel in traumaticin (gutta-percha 1, chloroform 9) acts favorably. He had tried the method in fourteen cases, in many of which inunctions, injections, etc., had failed.—*M. für Prak. Derm.*, XIX., No. 5.

### DOUBLE SYPHILITIC INFECTION.

Servin (*Presse Med. Belg.*, 1894, No. 6) reports two cases. The first shows simultaneously a chancre on the lower lip and upon the nipple, with involvement of the submaxillary and axillary glands. The genitals were not affected.

The second case showed simultaneous infection of the lower lip and the gum.—*M. für Prak. Derm.*, XIX., No. 5.

### SYPHILITIC RE-INFECTION.

Milligan (*Presse Med. Belg.*, 1894, No. 6) reports the case of a man of thirty-two. Ten years before he was treated for chancre of the finger, and had the secondary symptoms. The second affection was a "chancre-like" ulcer on the ala nasi which healed in three weeks. Seven weeks later "a roseola."—*M. für Prak. Derm.*, XIX., No. 5.

[Cases of re-infection cannot be proven without the absolute exclusion of other skin diseases and of the late manifestations of syphilis. Many reported cases are "not proven."—ED.]

---

### CHANCRE OF THE HAND OR FINGER.

A. Fournier (*Joun. des Mal. Cut. et de Syph.*, 1893, p. 678) says these occur rarely. Of ten thousand chancres, only forty-nine were upon the hand or finger, but the proportion might be greater since the nature of lesions there is often not recognized. The finger chancre is more often seated near the nail. Clinically there are some variations from the penile chancre, the most marked being the pain if the lesion is about the nail. The prognosis in finger chancres is rendered less favorable because of the frequency of its late recognition, and the physician so often treats it imperfectly and does not give it sufficient protection.—*M. für Prak. Derm.*, XIX., No. 5.

[The above recalls the case of a man who attempted to perform the duties of an accoucheur for the wife of a man who then had a sore on his penis. He got a chancre over the second phalanx of the right middle finger; later the papular syphilitic eruption, and involvement of the glands. The diagnosis was not made without the history in this case.—ED.]

---

### THE NEW YORK DERMATOLOGICAL SOCIETY.

234TH REGULAR MEETING. HELD ON TUESDAY EVENING, APRIL 24, 1894.

*Dr. C. W. Allen, President, in the Chair.*

*A Case of Nœvus of the Lip.*—Presented by Dr. J. A. Fordyce. The patient was a child aged two years, with a cavernous tumor of the lower lip, which extended for some distance into the mucous membrane, and ended at the margin of the skin and the mucous membrane. During the past few months it had enlarged quite rapidly. Dr. Fordyce said his idea was to treat the lesion by galvano-puncture or by electrolysis. The child also had a small nævus on one ear.

Drs. Bulkley, Taylor, Elliot, and Allen were of the opinion that such cases should be referred to the surgeon for excision, Dr. Elliot defining them as cavernous angiomata.

Drs. Piffard, Jackson, and Cutler stated that treatment by electrolysis or galvano-puncture was not very successful, Dr. Piffard declaring, that from reading the treatment always appeared satisfactory, but from actual experience was not. Dr. Allen reported a case of nævus of the lip extending into the mucous membrane, as cured by electrolysis, but it left a scar. The case of nævus of the vulva previously presented to the Society had greatly improved under electrolysis.

Drs. Taylor and Keyes had obtained good results from piercing them with a curved shoemaker's awl heated to white heat.

Dr. Sherwell recommended for the operation of nævi on the lip the use of a large lid clamp, which he had found very serviceable in the application of the galvano-cautery in two cases of nævi of the eyelid, treated by him recently with very good results.

Dr. Piffard mentioned that he and Dr. Keyes once dissected out a supposed nævus from the arm and found it to be a sarcoma.

*A Case for Diagnosis.*—Presented by Dr. Fordyce.

The patient was a child with two lesions, about the size of a silver dime, one on each buttock. They were symmetrically located, and both were covered by a scab. Upon the removal of one of the scabs a granulating surface was found underneath. The mother stated that these lesions had been present for six months, and were gradually becoming larger. There was no history of syphilis; there were no other lesions on the body. Dr. Fordyce said he thought the diagnosis lay between lupus and syphilis, although the lesions did not present very pronounced features of either disease.

Drs. Elliot, Cutler, Jackson, and Lustgarten made the diagnosis of granuloma from local infection. In reply to a question Dr. Lustgarten said that the term infectious granuloma had a wide denomination, meaning granulation tissue of known or supposed infectious nature. If we did not know the exact etiology of such a lesion, we might call it an infectious granuloma, leaving open the question if it was tubercular, syphilitic or of other nature.

Dr. Fordyce said he was inclined to regard the case as one of lupus, on account of the long duration of the lesions, and their appearance underneath the crust.

*A Case of Psoriasis.*—Presented by Dr. Fordyce.

The patient was a woman with peculiar depressed squamous lesions covering both arms and forearms and both knees, which had existed for a number of years. The case was undoubtedly one of psoriasis, but unusual on account of the marked redness and depression of the lesions. On the knees the psoriatic lesions were quite characteristic. Some of the lesions presented well-marked fissures.

Dr. Klotz said that some of the lesions, which did not show the clear features of psoriasis, were probably the seat of an artificial dermatitis, resulting from the application of carbolic acid.

*A Case of Warts.*—Presented by Dr. Piffard for Dr. A. R. Robinson.

The patient was a woman with an extraordinary development of warts covering the palmar and dorsal aspect of both hands. These had existed for five years. They were gradually becoming flatter. The patient also had a few warts on the face.

Dr. Klotz inquired what results had been obtained by the members present from treatment, as he had found the remedies usually recommended quite ineffective, particularly salicylic acid.

Dr. Jackson thought the case served to illustrate the infectious nature of warts. Dr. Lustgarten said that the large number of the warts and the manner of spreading suggested auto-inoculation. He recommended a thirty per cent. alcoholic solution of salicylic acid; so did Dr. Sherwell, Dr. Jackson salicylic acid plaster.

Drs. Piffard, Keyes, and Cutler reported favorable results from the external and internal use of the tincture of thuja. Dr. Piffard employs a strong tincture, beginning with five drops and running up to a dram, three times a day. In some cases the drug proved very efficacious, while in others it had failed absolutely; it had been known to give rise to dermatitis and phlebitis. Dr. Keyes had given the tincture of thuja in tablespoonful doses; in some cases of papillomata it had undoubtedly effected a cure. Dr. Cutler reported a case in which probably over one thousand warts had been present on different parts of the body. Under the use of thuja both externally and internally, they had almost entirely disappeared.

Dr. Allen called attention to the fact that this patient, as she informed him, had taken a great deal of arsenic, apparently without much effect.

*A Case for Diagnosis.*—Presented by Dr. Cutler.

The patient was a young woman with lesions on the tip of the nose, and on both palms. The lesions on the palms commenced about one year ago as a thickening of the skin, which gradually became worse. Unless she constantly employed some form of ointment the skin became fissured. The lesions on the nose first appeared about five months ago and are gradually enlarging. They consist of papules covered with yellowish crusts surrounded by a zone of redness. The patient appeared to be otherwise healthy, and presented no symptoms of constitutional trouble. There were no lesions on the feet. The fingers were gradually becoming contracted.

Drs. Lustgarten, Sherwell, Fordyce, Bulkley, and Klotz were of the opinion that the lesions on the palms and that on the nose were entirely distinct; the affection on the palms was a tylosis, probably due to the patient's occupation or washing the hands with alkalies, etc. The lesions of the nose was considered to be lupus erythematosus.

Dr. Bulkley thought the affection on the nose was a superficial process which would readily disappear under local treatment. Dr. Fordyce mentioned the marked thickening of the horney layer of the skin. Dr. Sherwell observed that he found great improvement by the use of salicylic acid in solution in a case of tylosis of the palms and heels.

Dr. Jackson diagnosed the case as one of chronic eczema of the palms and perhaps also of the nose.

Dr. Elliot diagnosed the case as one of seborrhoic eczema of the palms of the hands and of the nose.

Dr. Cutler said he was inclined to believe that the nose lesion was either lupus or syphilis—probably syphilis. He could arrive at no satisfactory diagnosis regarding the lesions on the hands. He did not believe they were due to the patient's occupation, for she had been doing the same kind of work for many years and the lesions first appeared about one year ago, and had gradually been getting worse during the past two or three months. The hands were usually absolutely dry. He would place the patient at once on antisyphilitic treatment and report at the next meeting.—*Journal of Cutaneous and Genito-Urinary Diseases,* October 1894. Part of report.

